# Local assessment of the immunohistochemical expression of Foxp3^+^ regulatory T lymphocytes in the different pathological forms associated with bovine paratuberculosis

**DOI:** 10.1186/s12917-022-03399-x

**Published:** 2022-08-04

**Authors:** David Zapico, José Espinosa, Miguel Fernández, Miguel Criado, Noive Arteche-Villasol, Valentín Pérez

**Affiliations:** Departamento de Sanidad Animal, Facultad de Veterinaria, Instituto de Ganadería de Montaña (CSIC-ULE), Universidad de León, C/ Profesor Pedro Cármenes s/n, E-24071 León, Spain

**Keywords:** *Mycobacterium avium* subsp, *paratuberculosis*, Foxp3^+^, intestinal tissue, type of lesion, regulatory T lymphocytes, cattle

## Abstract

**Background:**

*Mycobacterium avium* subsp. *paratuberculosis* infected animals show a variety of granulomatous lesions, from focal forms with well-demarcated granulomas restricted to the gut-associated lymphoid tissue (GALT), that are seen in the initial phases or latency stages, to a diffuse granulomatous enteritis, with abundant (multibacillary) or scant (paucibacillary) bacteria, seen in clinical stages. Factors that determine the response to the infection, responsible for the occurrence of the different types of lesion, are still not fully determined. It has been seen that regulatory T cells (Treg) play an important role in various diseases where they act on the limitation of the immunopathology associated with the immune response. In the case of paratuberculosis (PTB) the role of Treg lymphocytes in the immunity against Map is far away to be completely understood; therefore, several studies addressing this subject have appeared recently. The aim of this work was to assess, by immunohistochemical methods, the presence of Foxp3^+^ T lymphocytes in intestinal samples with different types of lesions seen in cows with PTB.

**Methods:**

Intestinal samples of twenty cows showing the different pathological forms of PTB were evaluated: uninfected controls (*n* = 5), focal lesions (*n* = 5), diffuse paucibacillary (*n* = 5) and diffuse multibacillary (*n* = 5) forms. Foxp3^+^ lymphocyte distribution was assessed by differential cell count in intestinal lamina propria (LP), gut-associated lymphoid tissue (GALT) and mesenteric lymph node (MLN).

**Results:**

A significant increase in the number of Foxp3^+^ T cells was observed in infected animals with respect to control group, regardless of the type of lesion. However, when the different categories of lesion were analyzed independently, all individuals with PTB lesions showed an increase in the amount of Foxp3^+^ T lymphocytes compared to the control group but this increase was only significant in cows with focal lesions and, to a lesser extent, in animals with diffuse paucibacillary forms. The former showed the highest numbers, significantly different from those found in cows with diffuse lesions, where no differences were noted between the two forms. No specific distribution pattern was observed within the granulomatous lesions in any of the groups.

**Conclusions:**

The increase of Foxp3^+^ T cells in focal forms, that have been associated with latency or resistance to infection, suggest an anti-inflammatory action of these cells at these stages, helping to prevent exacerbation of the inflammatory response, as occurs in diffuse forms, responsible for the appearance of clinical signs.

## Background

The immune system has developed relevant regulatory mechanisms to prevent the exacerbated responses that can be the consequence of autoimmunity and immunopathology. Among them, there is a population of regulatory T cells (Treg), a heterogeneous groups of T lymphocytes involved in the downregulation of immune responses during the progression of chronic diseases, with the aim of reducing potentially harmful effects of the immune response to the host, such as immune-mediated pathology, or by facilitating immune evasion by pathogens causing chronic infections [1, 2]. This group of cells shows different phenotypes that trigger a variety of inhibitory mechanisms and functions. The predominant Treg types are CD4^+^ and express either or both CD25 and the forkhead box transcription factor, Foxp3 [3, 4]. Foxp3 currently represents the most specific marker used to distinguish regulatory cells (Foxp3^+^CD25^+^CD4^+^) from activated effector cells (Foxp3^−^CD25^+^CD4^+^) within the CD25^+^CD4^+^ cell subpopulation [5, 6].

Treg lymphocytes have been widely recognized in humans and mice [7] and different studies have been conducted in other species in order to elucidate their functions in specific diseases. In this way, it has been seen that these cells play an important role in the progression and development of various viral [8], bacterial [9, 10] or parasitic diseases [11, 12] where they are related to a limitation of the immunopathology associated with the immune response. However, studies related to Treg lymphocytes in cattle are relatively rare. In the case of paratuberculosis (PTB), a chronic wasting disease of ruminants caused by *Mycobacterium avium* subspecies *paratuberculosis* (Map), the role of Treg lymphocytes in the immunity against Map has been investigated in some studies, but currently is far to be fully understood [13–17].

Map-infected animals display a long subclinical stage characterized by the development of an effective Th1 pro-inflammatory immune response to Map antigens that would be able to control intracellular infection. Late subclinical disease or near the transition from subclinical to clinical disease, when the animals show diarrhea and premature death, is characterized by the predominance of a non-protective Th2 immune response [18, 19]. Some recent studies indicate that immune responses against Map infection are more complex and could go beyond the Th1 to Th2 transit, with a combined response where both antibody and cellular immunity may play key roles in the disease manifestation or resistance [20]. At present, it is not well known why the Th1-to-Th2 immune balance shifts away from an effective Th1 response towards an unproductive Th2 response. In animals able to control the infection, it is hypothesized that a population of Treg cells could appear in response to the chronic low stimulation with Map antigen, limiting the appearance of an uncontrolled and ineffective cellular Th1 immune response during subclinical infection, thus preventing the development and progression of more severe lesions [21]. In this sense, there are studies showing that stimulation of peripheral blood mononuclear cells (PBMCs) from Map-infected cows, with Map, causes increases of IL-10 or TGF-β expression compared to non-infected animals [22, 23]. These anti-inflammatory and regulatory cytokines, although also produced by macrophages, are largely released by CD4^+^CD25^+^Foxp3^+^cells, which in turn are increased in Map-infected animals [14, 24, 25]. In addition, increases in gamma delta (γδ) and Foxp3^+^ regulatory T cell subsets have also been observed both in peripheral blood and at the local level in cows with PTB infection [13–17, 26].

All these evidences support the hypothesis that regulatory T cell populations would play an important role in the pathogenesis of PTB. Previous reports have shown that there is a close relationship between the type of lesion that the infected animals develop and their clinical status, from focal lesions, restricted to the intestinal lymphoid tissue, associated with the containment of the infection, to diffuse granulomatous lesions, seen in advances stages [27, 28], and where different subpopulations of regulatory T cells would play a crucial role in the development of these different pathological forms [13, 15, 29]. However, there is hardly any information on the presence and distribution of these cells in relation to the specific granulomatous lesions associated with Map infection. At present, the existing studies that analyze the role of Foxp3^+^ lymphocytes in bovine PTB differ in terms of the methodology used, and the presence and levels of these cells have not been fully evaluated among the different pathological forms associated with the disease [14, 15, 17]. The information available on the expression of the Foxp3^+^ T cells at the intestinal level is mainly focused on the lesions associated with the clinical course of the disease, with scarce data of these cells regarding those related to the subclinical stages [14, 15, 17].

In the present study, we analyze the expression of Foxp3^+^ regulatory T cells, through quantitative evaluation of immunolabeled cells, in intestinal tissue samples taken from cattle naturally infected with Map. For this purpose, we evaluated the amount and distribution of Foxp3^+^ T cells using immunohistochemical methods, in cows showing focal granulomatous lesions (subclinical forms related to latency or resistance), diffuse granulomatous enteritis (associated with clinical stages) and non-infected healthy cattle. This knowledge will improve the understanding of the biology of these cells and the immunopathology of PTB.

## Results

**Fig. 1:**
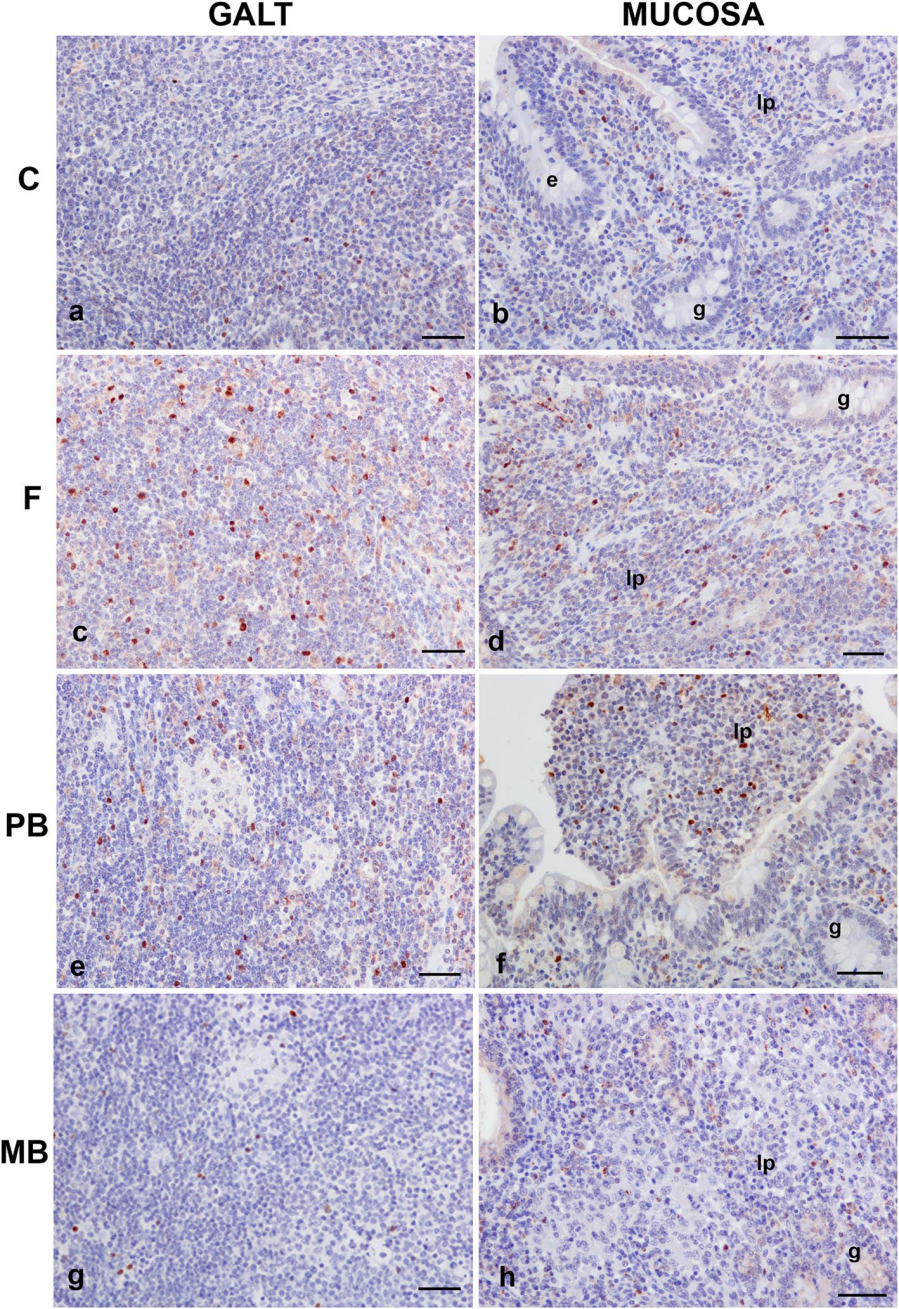
Photomicrographs of jejunal tissue samples showing immunolabelling for Foxp3^+^ T cells, from control (*a-b*) and infected animals (*c-h*) showing different types of lesions associated with Map infection. (*a-b*). Positively immunolabelled cells show a bright brown stain in their cytoplasm. Control (C) animals with low presence of Foxp3^+^ T cells both in gut-associated lymphoid tissue (GALT) (*a*) and intestinal mucosa (MUCOSA) (*b*). (*c-d*) Animals with focal (F) lesions with a pronounced increase of immunolabelled Foxp3^+^ T lymphocytes is observed in the GALT (*c*) and to a lesser extent in the lamina propria (*d*). (*e-f)* Diffuse paucibacillary (PB) lesions where Foxp3^+^ T lymphocytes are also seen among the lymphocytic infiltrate of the LP (*f*) and in higher numbers on the GALT (*e*). (*g-h*) Diffuse multibacillary (MB) lesions with reduced presence of Foxp3^+^ T cells either in the GALT (*g*) or among the granulomas of the lamina propria (*h*), in similar amounts that the control (C) group. No specific distribution patter of the Foxp3^+^ T cells were detected in relation with the granulomas in any intestinal areas analyzed. Scale bar = 200 μm. Harris´s haematoxylin counterstain. e: epithelium lining the intestinal mucosa; lp: lamina propria; g: intestinal glands.

Positively immunolabelled Foxp3^+^ T cells were identified by the presence of a fine brown-colored cytoplasmatic staining. All of them showed a round central nucleus with scant cytoplasm and were morphologically consistent with lymphocytes. They were identified in all samples analyzed, from both infected and non-infected cows (Fig. 1).

According to our model selection procedure, the most strong and parsimonious model, based on the fit quality indicators AIC (model with substantial support: ∆AICc < 2 unit given the data set) and Wi (Akaike weight of the model) (Table 1), included an additive combination and its interaction of all explanatory variables considered. Estimation of the adjusted R^2^ of the fitted model explained 51.30 % of the variability observed in the number of positively immunolabelled Foxp3^+^ T cells (Table 1). In turn, inter-individual differences contributed a 34.37 % to the variance in the Foxp3^+^ cell number.

**Table 1:** Linear mixed model selection for the effects of: infection status (IS), intestinal area (IA) and type of lesion (TL) on the number of positively immunolabelled Foxp3^+^ T cells.

Biological models	AIC_c_	∆AIC_c_	AIC_c_W_i_	R^2^ (adj)
IS+TL+IA+IA*TL	1136.42	0.00	0.487	0.513
IS+IA+TL	1145.23	8.81	0.323	0.503
IA*TL	1172.21	35.79	0.126	0.355
IA+TL	1198.91	62.42	0.059	0.322
Null model	1213.43	77.01	0.004	0.012

Note: Models with substantial support are in bold.

* : interaction, + : additive effect, AIC_c_: Akaike's information criterion corrected for small sample size, ΔAIC_c_ : difference of AICc between the model and the most parsimonious model; AIC_c_W_i_ : Akaike weight of the model, *R*^*2*^(adj) = adjusted *R*^*2*^ of the fitted model.

**Fig. 2:**
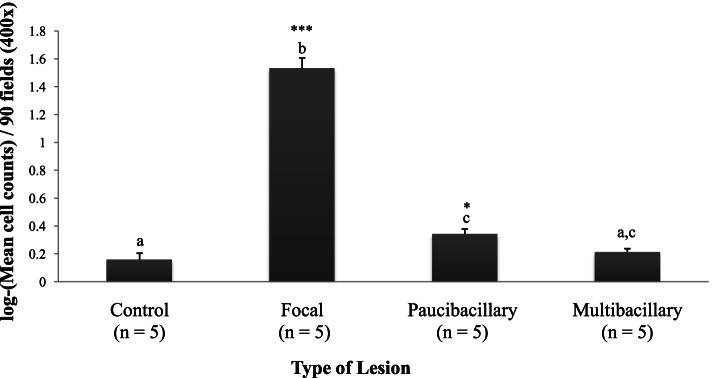
Bar-plot that shows the total mean cell counts and standard error (log-transformed) of positively immunolabelled Foxp3^+^ T cells subset according to the type of lesion. Different superscript letters indicate statistical significance between the different type of lesion. ^***^ *p* < 0.001; ^*^ *p* < 0.05. Specific *p*-value resulting from each pairwise comparison between lesion category groups can be consulted in the Table 2.

Results of the *post-hoc* analysis and *p*-values are showed in Table 2. The mean number (log-transformed) of Foxp3^+^ lymphocytes varied significantly (*p* < 0.001) between non-infected animals (0.19 ± 0.033) and Map-infected cattle (0.71 ± 0.012), regardless of the type of lesion. Similarly, these values showed significant differences when the different categories of lesion were analyzed (Fig. 2). All individuals with PTB lesions showed an increase in the amount of Foxp3^+^ T lymphocytes compared to the control group. However, this increase was only significant in cows with focal lesions (*p* < 0.001), and animals with diffuse paucibacillary forms (*p* < 0.05) (Table 2). The highest number of these cells was detected in cows with focal lesions, showing significant differences (*p* < 0.001) with respect to the group with diffuse forms. No differences were detected in the number of positively immunolabelled Foxp3^+^ T lymphocytes between individuals with diffuse paucibacillary and those with multibacillary lesions (*p* = 0.686).

**Table 2:** Results of the *post-hoc* Tukey’s Honestly Significant Difference test for all pairwise comparisons in the model classified with ∆AICC < 2 for the number of positively immunolabelled Foxp3^+^ T cells.

Linear Hypotheses	Estimate	SE	Z-value	*p*-Value
*Infection status (IS)*				
IS (H) — IS (I) = 0	-0.201	0.024	- 8.144	*p* < 0.001
*Type of lesion (TL)*				
TL (C) — TL (F) = 0	- 0.551	0.024	- 22.132	*p* < 0.001
TL (C) — TL (DP) = 0	-0.192	0.026	-3.45	*p* < 0.05
TL (C) — TL (DM) = 0	-0.081	0.016	-0.342	*p* = 0.986
TL (F) — TL (DP) = 0	0.532	0.012	20.401	*p* < 0.001
TL (F) — TL (DM) = 0	0.561	0.035	22.92	*p* < 0.001
TL (DP) — TL (DM) = 0	0.028	0.011	1.105	*p* = 0.686
*Intestinal area (IA)*				
IA (LP) — IA (GALT) = 0	-0.171	0.025	-3.221	*p* < 0.01
IA (LP) — IA (MLN) = 0	-0.126	0.027	-1.178	*p* < 0.01
IA (GALT) — IA (MLN) = 0	0.114	0.016	4.142	*p* = 0.466
Type of lesion (TL)* Intestinal area (IA)				
TL (C)*IA (LP) — TL (C)* IA (GALT) = 0	-0.076	0.042	-0181	*p* > 0.100
TL (C)*IA (LP) — TL (C)* IA (MLN) = 0	-0.039	0.044	-0.870	*p* > 0.100
TL (C)*IA (GALT) — TL (C)* IA (MLN) = 0	-0.047	0.024	-1.107	*p* > 0.100
TL (F)*IA (LP) — TL (F)* IA (GALT) = 0	-0.321	0.038	-7.881	*p* < 0.001
TL (F)*IA (LP) — TL (F)* IA (MLN) = 0	-0.162	0.021	-1.265	*p* = 0.016
TL (F)*IA (GALT) — TL (F)* IA (MLN) = 0	-0.239	0.047	-6.651	*p* < 0.01
TL (DP)*IA (LP) — TL (DP)* IA (GALT) = 0	-0.181	0.044	-1.761	*p* < 0.05
TL (DP)*IA (LP) — TL (DP)* IA (MLN) = 0	-0.082	0.012	-1.702	*p* < 0.05
TL (DP)*IA (GALT) — TL (DP)* IA (MLN) = 0	0.035	0.045	0.742	*p* > 0.05
TL (DM)*IA (LP) — TL (DM)* IA (GALT) = 0	-0.024	0.040	-0.621	*p* > 0.100
TL (DM)*IA (LP) — TL (DM)* IA (MLN) = 0	-0.019	0.043	-0.602	*p* > 0.100
TL (DM)*IA (GALT) — TL (DM)* IA (MLN) = 0	-0.011	0.025	-0.164	*p* > 0.100

**Fig. 3:**
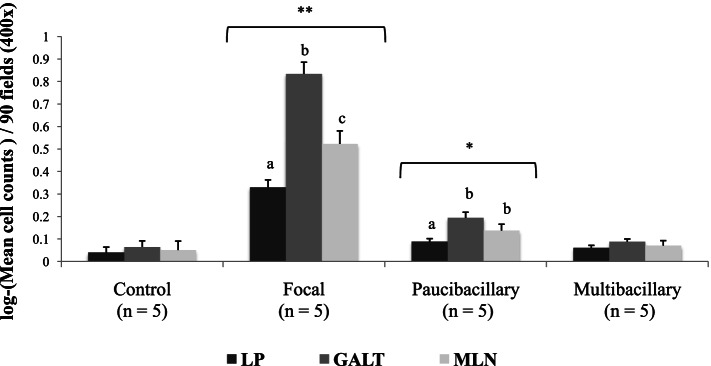
Bar-plot that shows the mean cell counts and standard errors (log-transformed) of positively immunolabelled Foxp3^+^ T cell subset in the different intestinal areas analyzed according to the type of lesion. LP: Lamina propria; GALT: gut-associated lymphoid tissue; MLN: mesenteric lymph node. Different superscript letters indicate statistical significance between the different intestinal areas within each type of lesion. ^**^ *p* < 0.01; ^*^ *p* < 0.05.Specific *p*-value resulting from each pairwise comparison between intestinal areas within each type of lesion can be consulted in the Table 2.

SE: standard error, *: interaction, H: healthy, I: infected, C: control, F: Focal, DM: diffuse paucibacillary, DP: diffuse multibacillary, LP: Lamina propria, GALT: gut-associated lymphoid tissue; MLN: mesenteric lymph node.

Regarding the counting of immunolabelled Foxp3^+^ T lymphocytes according to the different intestinal areas analyzed, a heterogeneous distribution was observed, with the greatest number of positively marked cells observed in the gut-associated lymphoid tissue (GALT) followed by the mesenteric lymph node (MLN) and, in a less amount, the lamina propria (LP), regardless of the type of lesion (Fig. 3) (Table 2). However, this variation was only statistically significant in individuals with focal and diffuse paucibacillary lesions (Table 2). Finally, in all the animals of the study, it was observed that the increase in Foxp3^+^ T lymphocytes present in the GALT was significantly associated with a greater number of these cells in the regional MLN (r_t_ = 0.47, *p* < 0.001). Similarly, although to lesser extent, a slight increase in the amount of these cells was observed at the LP level in animals that showed a higher number of these lymphocytes at the intestinal lymphoid tissue level (MLN and GALT) (r_t_ = 0.21, *p* < 0.01).

With respect to the distribution of positively immunolabelled Foxp3^+^ T lymphocytes according to the granulomatous infiltrate associated with the different types of PTB lesions, no specific distribution pattern was observed in any of the pathological form evaluated. In some cases, positively marked Foxp3^+^ T lymphocytes were more numerous in the vicinity of the granulomatous infiltrate while in others, were disposed in a widespread manner, with no differences between those areas in which the granulomatous infiltrate was present, from other zones of the intestine devoid of granulomatous infiltrates (Fig. 1). This non-specific distribution pattern was consistently observed in each of the intestinal areas evaluated.

## Discussion

This study has evaluated, by immunohistochemical labelling, the presence and distribution of Foxp3^+^ T lymphocytes in relation to the different types of granulomatous lesions associated with bovine PTB. Although there are previous works that analyze the possible role of these cells in paratuberculosis [14, 15, 17], the present study has the novelty to evaluate the presence and distribution of Foxp3^+^ T lymphocytes in the main pathological forms associated with the Map infection, both in individuals with focal lesions, animal with diffuse granulomatous lesions and non-infected animals, by their differential assessment in all the different areas of the jejunum (LP and GALT), as well as the regional MLN. In addition, the methodology used in this study shows important differences, mainly in relation to the total number of animals analyzed or classification of the different types of lesions. The results of the present work show that variations in the amount of this particular type of T lymphocytes are seen in the different types of lesion found in PTB, and its role should be further clarified. Recently, it has been evidenced that Map-infected cows show higher levels of PBMCs with CD4^+^ CD25^+^ Foxp3^+^ phenotype than control animals [14, 24]. This indicates that infection with this mycobacterium is an important stimulus for the release of this cell subtype through the production and release of specific cytokines, and that there could be a correlation between the Foxp3^+^ T lymphocytes levels at the peripheral blood and those existing at the intestinal tissue.

Regulatory T cells have been regarded as a crucial element in the progression of PTB in ruminants in previous studies [16, 21, 24, 30]. Evidence has shown that Treg cells would not play a role in establishing primary infection [31] but rather, may facilitate the shift between Th1 and Th2 immune responses [21, 24] indicative of the transition from subclinical to clinical disease. In this line, one interesting result of this study was the fact that the highest numbers of positively immunolabelled Foxp3^+^ T cells were observed in animals with focal lesions, in comparison to the rest of the groups. These lesions are characterized by the presence of small granulomas located exclusively at the GALT and have been associated with persistent or latent subclinical infections in which animals are able to contain Map infection at this level, controlling its progression [27, 28, 32, 33]. Thus, it seems appropriate to consider the hypothesis that these cells could play a role in the prevention of lesions from progressing from the GALT to the LP, participating in the regulation of the initial Th1 type pro-inflammatory response and therefore the maintenance of immune homeostasis. In this sense, it has been seen that cows with clinical signs, associated with diffuse lesions, show lower levels of Foxp3^+^ cells at the intestinal level than uninfected animals [15, 34]. Nevertheless, opposing conclusions on the role of these cellular subtypes have been reached in other diseases, such as tuberculosis or various parasitic infections. Experimental studies on mice infected with *Mycobacterium tuberculosis* showed that the depletion of Foxp3^+^ cells in tuberculous lung lesions promoted a pro-inflammatory action, with a reduction in the bacterial load associated with a lower local inflammatory response [10]. On the other hand, other studies observed local increases of these cells in early stages of infection by different parasitic agents and then, in chronic and severe lesions [12, 35, 36]. These findings suggest a wide functional variability of this cell subtype according to the type of pathogen involved and that they could show a dual role: minimizing the tissue pathology and the clinical picture and, on the other hand, modulate the host immune response to facilitate the pathogen survival. In the present study, a lower number of Foxp3^+^ T lymphocyte levels was observed in animals with diffuse lesions, both paucibacillary and multibacillary, characterized by a severe granulomatous inflammatory response that affects wide areas of the intestine. This result could lead to the hypothesis that reduced levels of these cells would favor the intense inflammatory response seen in the advanced phases of the disease. .

In our study, we did not identify specific distribution patterns of positively immunolabelled Foxp3^+^ T cells around intestinal granulomatous lesions in any of the pathological forms analyzed, unlike that observed in other conditions [10, 12, 35, 36], or as in other regulatory T cells subsets previously analyzed [16]. However, despite not showing a specific lesion distribution, an interesting result was that the highest number of Foxp3^+^ T lymphocytes was observed in GALT, followed by the regional MLN, significantly higher in animals with focal lesions. This could indicate that, in these areas, where granulomatous lesions are concentrated in the focal forms, a high differentiation of Treg cells takes place, triggered by the antigenic stimulation of Map, suggesting an involvement of these cells in the containment of granulomatous lesions. In contrast, at the LP level, where the lesions progress in diffuse forms, the number of positively immunolabelled Foxp3^+^ T cells observed was very low. This suggests a suppression or destruction of these cells in this intestinal area caused by other regulatory T subsets [29, 37], by specific cytokines, such as IL-10 or TGF-β, produced by certain of helper T lymphocytes subsets [38], by the macrophages that make up the diffuse granulomatous infiltrate present in LP [39, 40] or by the Foxp3^+^ T cells themselves [22]. Another aspect that must be taken into consideration is that the jejunal lymphoid tissue, unlike the LP, and without the need of antigenic stimulation by Map, is an important immune induction site, where large numbers of immune cells are recruited, including Treg cells. This fact may influence the variability observed in the number of Foxp3^+^ cells between GALT and LP, regardless of type of lesion. In this sense, and taking into account the underlying biology of the intestinal environment, unique immune cell landscapes in each of these intestinal compartments could contribute to the different types of lesion observed. However, based on the results obtained in our study, the presence of Map also seems to stimulate an increase in the number of Foxp3^+^ cells in GALT, since a greater number of these cells was observed in the lymphoid tissue of all infected individuals in comparison to the control group.

These results will serve to continue expanding the knowledge about the development of lesions and the establishment of the local immune response against Map, which seems to play a key role in whether animals achieve containment of the infection in granulomas in the lymphoid tissue, or these evolve into a diffuse granulomatous enteritis associated with clinical forms. Additional studies would be necessary to better understand the role of Foxp3^+^ T cells in Map infection, together with the interactions with other lymphocyte subsets or Map-infected macrophages, as well as their cytokine expression, in the different types of lesions. In turn, the existence of differential host immune responses against Map according to the different affected intestinal areas (for example, the jejunum and ileum) [41–43] make additional studies necessary to confirm whether or not our findings can be extrapolated to other anatomical intestinal sites. This would allow to advance in the knowledge on the host-Map interactions and the identification of the immune responses associated with the control or progression of this mycobacterial infection in the natural host, with the investigation of the causes beyond the low presence of Foxp3^+^ T cells in animals with diffuse lesions and therefore increasing the knowledge on PTB pathogenesis.

## Conclusions

The presence at the intestinal level of a greater number of Foxp3^+^ T lymphocytes in infected cows and, especially in those with focal lesions, could suggest that these cells would participate in an early, specific and possibly direct immune response developed against Map. In addition, the higher number of Foxp3^+^ T cells in the GALT of cows with focal lesions, when compared to animals with diffuse forms, suggest a possible protective role of this lymphocyte subset. Its action could be the participation on the containment of Map-infection at the level of lymphoid tissue, favoring its latency and avoiding the exacerbated inflammatory response responsible for the clinical picture observed in the diffuse generalized forms. Considering this premise, it might be possible to achieve some beneficial outcomes by stimulating the different Treg cell subsets involved in Map infection, especially during immunization.

## Material and methods

### Animals and experimental design

Initially, for this study, a total of 30 Holstein Friesian cows (ranging from three and six years-old) were analyzed. They belonged to a commercial dairy herd in which a follow-up survey on losses due to PTB was ongoing. Animals were culled in an authorized slaughterhouse following the standard methods in the current legislation. Samples from different areas of the intestine and mesenteric lymph nodes were fixed in 10 % buffered formalin of stored at -20 °C. Infection was confirmed in 25 of them both by bacteriological culture of frozen intestinal tissues and nested-PCR, using primers to detect the presence of Map-specific IS*900* DNA, and following the methodology detailed elsewhere [28, 44]. Five samples were negative and used as uninfected controls in the study.

### Tissue samples and classification of paratuberculosis lesions

Samples from ileocecal valve, ileum, jejunum (with Peyer´s patches) and associated mesenteric lymph nodes were taken from each animal for microbiological, histopathological and molecular analyses. After fixation in 10 % buffered formalin, they were routinely embedded in paraffin wax, after dehydration through a graded alcohol series and xylene treatment. Tissue sections 3-μm thick were obtained from each sample and stained with Harris´s haematoxylin and eosin (H&E) and Ziehl-Neelsen method for acid-fast bacilli (AFB) detection. A subjective classification of the section was made according to the amount of AFB present, from 0 (no detectable bacilli), 1 (scant AFB in the cytoplasm of macrophages), 2 (moderate, easily detectable bacilli), to 3 (high load of AFB).

No granulomatous lesion consistent with Map infection was observed in the five cattle that were negative for both bacteriological culture and nested-PCR tests, while specific lesions were detected in samples from the intestine and lymph nodes from the remaining animals. Subsequently, granulomatous lesions compatible with Map infection were classified into 3 categories -as focal, diffuse paucibacillary, and diffuse multibacillary-, according to the guidelines previously proposed [27]. Each animal was classified according to the most severe lesion found in the examined samples, bearing in mind that the type of lesion could vary among the tissue samples from the same individual.

Cows with focal lesions showed well defined granulomas, composed of small groups of macrophages, located exclusively in the interfollicular areas of the intestinal Peyer’s patches. They were also present in the cortical and paracortical areas of the mesenteric lymph nodes. Scant of none AFB were detected in these lesions (category 0 or 1). This type of lesion was identified in 10 infected cows. Positive results were obtained in tissues from five of these animals by bacteriological culture and nested-PCR against Map. In the remaining five, Map genetic material was detected by PCR, however they showed negative results by microbiological culture. Diffuse lesions were characterized by a widespread granulomatous infiltrate, present both in the lymphoid tissue and in areas of the lamina propria both related or not to the Peyer’s patches, and in the mesenteric lymph node. The infiltrate was formed by epithelioid macrophages, with some Langhans type giant cells. According the number of AFB present in the lesions they were classified as diffuse multibacillary forms, detected in 9 Map-infected animals, with a predominance of epithelioid cells in the infiltrate that harbor large amounts of AFB (category 2 o more frequently 3), of diffuse paucibacillary lesions, seen in 6 infected cows, where the infiltrate was formed by large amounts of lymphocytes with scattered groups of macrophages and giant cells, with reduced numbers of AFP present in their cytoplasm (score 1). All tissues with diffuse lesions, regardless of the intestinal section examined, showed positive results in both microbiological culture and nested-PCR test.

For the immunohistochemical analysis, five animals from each group, showing the most representative granulomatous changes from each type of lesion, were selected. In all of these tissue samples, both the microbiological culture and nested-PCR test showed positive results against the presence of Map.

### Immunohistochemical study

Immunohistochemical analysis was carried out in a total of 20 jejunal tissue sections (with lymphoid tissue) and other 20 jejunal lymph node samples, one from each animal finally included in the study. As stated, these sections were representative of the lesion category assigned to each animal. Tissue sections 3-μm thick were mounted on electro charged adhesive gelatin-coated microscope slides (Thermo Scientific, Waltham, MA, USA). For the detection of Foxp3^+^ T lymphocytes, a primary polyclonal antibody (rabbit IgG isotype) against bovine Foxp3 (NB100-39002; Novus Biologicals®, Centennial, USA), at a 1:150 dilution, was used. The trading house had reported through a verified customer review that this Foxp3 antibody showed reactivity in sections of formalin-fixed bovine tissues (unpublished data). Heat-mediated antigen retrieval was performed by means of PT Link® system, using pH 6.0 target retrieval solution (Dako-Agilent® technologies, Santa Clara, USA) for 20 min at 95°C. Immunohistochemical procedure was carried out as described elsewhere [16]. Appropriate species-and isotype-matched immunoglobulins were used as control. These included sections with an isotype control for the primary antibody, and the omission of the primary antibody.

### Cell counting

Due to the heterogeneous distribution of positively immunolabelled Foxp3^+^ T cells, a differential cell count was carried out on the LP, GALT and jejunal MLN from each sample included in the study. In each slide, 30 randomly chosen high-power fields (HPF, field visible at 400× magnification with a field number of the ocular = 22, size = 0.237 mm^2^), were selected from each of the three areas analyzed and photographed. (Nikon® Eclipse Ci microscope with Digital MD-E3-6-3 digital camera). The counting of positively immunolabelled cells was performed on digital images using the Cell Counting add-on of Image J program® (U.S National Institutes of Health, Bethesda, Maryland, USA). In total, 90 randomly HPF were evaluated from each animal analyzed. In each animal, the final value of cell count was obtained by calculating the average value for the 30 HPF of each intestinal area analyzed (LP, GALT and MLN) together with the average value of the 90 total HPF evaluated. Finally, in order to jointly assess the different types of lesion, the mean value between the five slides of each lesion type analyzed was calculated. A total of 21.33 mm^2^ of intestinal surface area was evaluated in each of the animals included in the study, 7.11 mm^2^ corresponding to each of the three intestinal areas analyzed. Additionally, the distribution of Foxp3^+^ T lymphocytes in relation to the presence of granulomas was evaluated, when these were found in each section.

Assessment of Foxp3^+^ T cells immunostaining was assessed independently by two of the authors (JE and VP, an ECVP-board certified pathologist), and discordant results were reviewed in a multiheaded microscope to reach consensus.

### Statistical analysis

We used linear mixed models (LMM) for longitudinal data analysis [45] to explore the effects of infection status (healthy or infected), intestinal area (LP, GALT and MLN) and type of lesion (control, focal, diffuse paucibacillary and diffuse multibacillary) on the number of positively immunolabelled Foxp3^+^ T cells at an individual level. In order to perform an appropriate fitting of the LMMs, we transformed the variables in the following way: the number of Foxp3^+^ T cells was the response variable and was log (x+1)-transformed, and infection status, intestinal area and type of lesion were the explanatory variables. In the models, the identity of each animal was included as a random factor in order to consider the inter-individual effects on the number of Foxp3^+^ T cells. Package “lme4” [46] was used to fit LMMs, with the “lmer” function. The *dredge*, *get.models* and *model.sel* functions included in the R package “MuMIn”, were employed to construct a set of candidate models with all the possible combinations of predictive variables and, according to them, we identified the best models using an automatic selection procedure based on the Akaike Information Criterion corrected for small sample sizes (AICc) provided by the *dredge* function [47]. The most strong and parsimonious model was selected using the combination of lower Akaike’s Information Criterion (AICc) and the Akaike weight (Wi). Subsequently, we estimated the Akaike weight (Wi) and the percentage of explained variability (R^2^) for each selected model [46]. The fit quality of the selected models was evaluated through the analysis of residual deviance, linearity, multicollinearity and overdispersion using diagnostic graphics (R packages “mctest” and “car”). Goodness of fit and estimation of the parameters of the model were evaluated by means of the approximation procedures of the Restricted Maximum Likelihood Estimation (REML). We also calculated between- and within-individual variability of the best selected models. Between-individual variation refers to the classic phenomenon of individual differences in a variable, and within-individual variation refers to variability from occasion to occasion within an individual. We estimated these variances using the *lmer* function in R package “lme4” [46]. Subsequently, to observe the differences between groups within the relevant variables included in the final fitted model, we used the Tukey’s Honestly Significant Difference adjustment for the whole pairwise comparisons using the *glht.function* with the “multcomp” package in R. We produced the bar-plots of the log-transformed dependent variable by groups of explanatory variable using the *ggplot* function in R package “ggplot2”. To observe the differences between groups within the relevant variables included in the final fitted model, we used the Tukey’s Honestly Significant Difference adjustment for the whole pairwise comparisons using the *glht.function* with the “multcomp” package in R. Finally, Pearson´s rank correlation test was applied to establish possible correlations between the number of Foxp3^+^ T cells present in each evaluated tissue area. *P*-values of less than 0.05 were considered statistically significant.

All statistical analyses were performed with the R software version 3.5.3 [48].

## Data Availability

The data supporting the conclusions of this article will be made available by the authors, under reasonable request.
